# A 52-Year-Old Woman Presenting With a Right Breast Mass: A Case Report

**DOI:** 10.7759/cureus.27179

**Published:** 2022-07-23

**Authors:** Robert P Frantz, Mariam Hanna

**Affiliations:** 1 Internal Medicine, Tulane University School of Medicine, New Orleans, USA; 2 Department of Radiology, University of Florida College of Medicine, Gainesville, USA

**Keywords:** magnetic resonance imaging breast, high risk breast cancer, breast cancer imaging, breast lesions and imaging modality, spindle cell metaplastic breast cancer

## Abstract

We present the case of a 52-year-old female who presented with a rapidly enlarging right breast mass. An initial breast ultrasound showed a solid mass with cystic components and posterior acoustical enhancement and mammography showed a mass in the same area without calcifications. Biopsy of the lesion revealed spindle cell carcinoma of the breast. To date, the patient has undergone radiation therapy and simple mastectomy and will receive post-operative radiotherapy in the coming months.

## Introduction

Breast cancer is the most common type of cancer in females in the world [1]. Of the various subtypes of breast cancer, metaplastic carcinoma is an uncommon, heterogeneous group of cancers that comprise 1% of invasive breast cancers [2]. Spindle cell carcinoma of the breast is a subtype of metaplastic carcinoma characterized on pathology by spindle cell morphology with epithelioid differentiation [3]. It is exceedingly rare and accounts for 0.02-0.5% of cases of invasive breast cancer in the world [4]. Clinically, most cases present with a rapidly growing, palpable mass in the breast, ranging in size from 2-15 cm [5,6].

Definitive diagnosis of spindle cell carcinoma of the breast is made histologically, but certain radiologic features can be utilized to move it higher on the differential diagnosis early in the diagnostic process. On ultrasound, the tumor most commonly presents as a lobulated, circumscribed mass with cystic parts and posterior acoustic enhancement [7]. Mammography typically shows an irregular mass with few cases showing evidence of calcification [8]. MRI findings typically include a round/oval solitary mass with central necrosis [4]. While rare, spindle cell carcinoma of the breast is an aggressive disease and prompt diagnosis and treatment is necessary to achieve optimal patient outcomes.

## Case presentation

A 52-year-old woman with a past medical history of fibrocystic breast disease presented with an enlarging mass in the upper outer quadrant of her right breast, which she noticed on self-examination. She initially attributed the mass to her fibrocystic breast disease but sought care after approximately six months of rapid growth. She reported breast pain associated with the mass but denied skin changes or nipple discharge. She had no personal history of cancer but did have a significant family history notable for breast cancer in her maternal and paternal grandmothers and paternal aunt. On physical exam, her breasts were asymmetric and there was a large, palpable mass in the upper outer quadrant of the right breast comprising two-thirds of the breast. There was no nipple eversion or axillary lymphadenopathy and there were no skin changes noted. She was subsequently referred for breast ultrasound and mammography.

**Figure 1:**
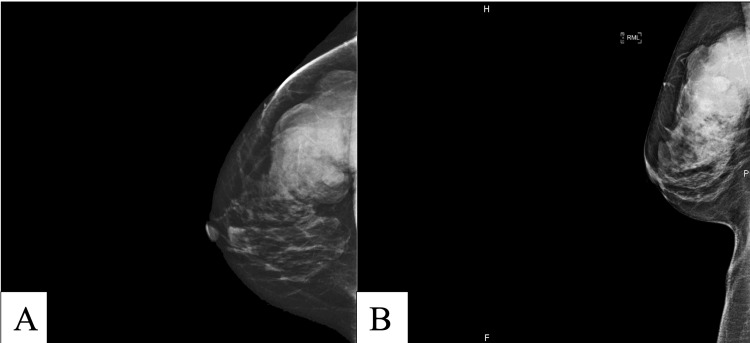
(A) Full paddle digital mammography craniocaudal view demonstrating large mass with lobulations in the lateral posterior aspect of the right breast. (B) Full paddle digital mediolateral oblique view mass is the upper posterior aspect of the right breast.

Ultrasound of the right breast showed a 7.5 cm mass at the 10:00 position of the right breast 8 cm from the nipple. It was predominantly solid with lobular margins and had cystic components and vascular flow. Some posterior acoustical enhancement was also noted. Mammography showed a 7.4 x 5.1 x 7.5 cm oval hyperdense circumscribed mass in the upper outer quadrant of the right breast (Figure 1). There was no evidence of calcification or axillary lymphadenopathy. Of note, a mammogram obtained five years prior showed no evidence of the current mass. A Breast Imaging Reporting and Data System (BI-RADS) score of 4 was assigned to her imaging and she was referred for tissue biopsy, which led to a diagnosis of malignant spindle cell carcinoma. The treatment plan included radiation to the breast followed by simple mastectomy and post-procedure radiation.

**Figure 2:**
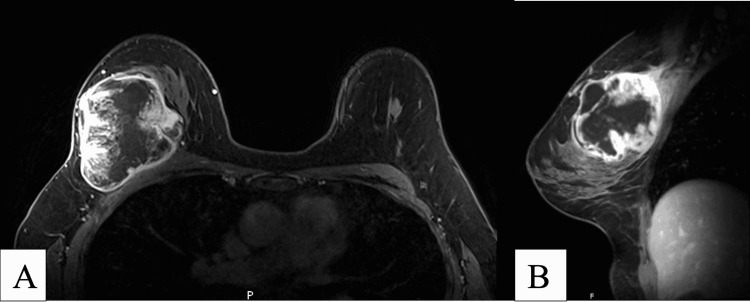
MRI performed prior to radiation therapy (A) Axial T1 MRI demonstrating heterogenous enhancement in the mass in the central outer aspect of the right breast with central necrosis; (B) Sagittal MRI images demonstrating large mass with heterogenous enhancement

**Figure 3:**
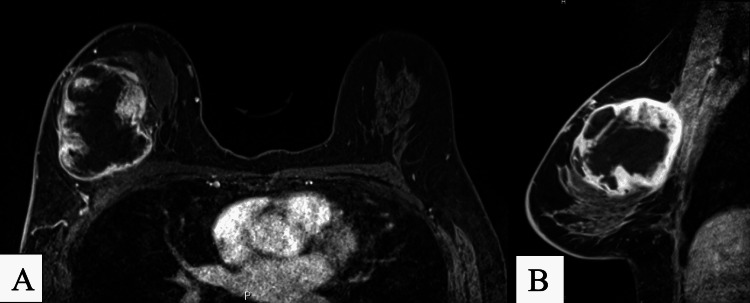
MRI performed following 25 cycles of radiation therapy (A) Axial T1 MRI demonstrating heterogenous enhancement in the mass in the central outer aspect of the right breast with central necrosis. Note the interval decrease in solid/peripheral enhancement; (B) Sagittal MRI demonstrating large mass with heterogenous enhancement interval decrease in solid component post treatment

Baseline CT and MRI was performed prior to beginning radiation treatment. MRI of the breast showed a 7.2 x 6 x 6.7 cm enhancing mass with T2 hyperintense non-enhancing areas consistent with necrosis, which corresponded to the biopsy-proven spindle cell carcinoma. The tumor was abutting the right pectoralis major muscle and post-contrast enhancement suggested tumoral involvement to the area (Figure 2). Initial CT scan of the chest showed no intrathoracic metastases. Following 25 rounds of radiation therapy, follow-up MRI and CT showed the mass was stable and the abnormal enhancement seen in the pectoralis major was no longer observed (Figure 3). The patient has since undergone mastectomy procedure and follow-up imaging will be obtained.

## Discussion

Spindle cell carcinoma of the breast is a rare, aggressive form of breast cancer that accounts for 0.02-0.5% of all cases of invasive breast cancer in women [4]. Due to the aggressive nature of the disease, prompt diagnosis and treatment is necessary to achieve optimal outcomes for patients. Patients will present with a breast mass in 82-100% of cases [7-9] and ultrasound and mammography are the initial steps in diagnosis. Biopsy of the mass will give the definitive diagnosis, but there are certain radiologic findings that can increase the level of suspicion for this disease early in the work-up of the patient.

Multiple studies have investigated the radiologic features of the disease among different imaging studies. Lai et al. [7] examined the sonographic features of these tumors and found that the lesion was circumscribed and gently lobulated in 90% of cases. They also showed that 70% of cases were either hypoechoic or very hypoechoic. All cases in the study performed by Lai et al. exhibited cystic areas and posterior acoustic enhancement. Alhaidary et al. [9] also reported posterior acoustic enhancement in 55% of cases. While this is not a specific finding for spindle cell carcinoma of the breast and can be seen in other breast masses [10], it is an important piece of information to narrow the differential diagnosis. In the case of our patient, ultrasound showed a mass with lobular margins with cystic components and posterior acoustic enhancement, which is consistent with what is reported in the literature. On mammography, the majority of cases are reported as masses with irregular shapes and indistinct margins [8,9]. Calcifications are not often seen on mammography. In the study performed by Langlands et al. [8] only 17% of cases exhibited this feature.

The MRI findings of spindle cell carcinoma of the breast were detailed in a series of 26 cases reported by Tokuda et al [4]. In this study, several radiologic features were common across the cases reported. Most masses were round with irregular margins. Additionally, 72% of cases exhibited rim enhancement and 96% showed washout kinetic analysis. Hyperintensity was seen on FST2WI in 84% of cases and on FST2WI and fast enhancing component on dynamic contrast enhanced in 56% of cases. A hypointense rim was seen on FST2WI in 72% of cases.

The 52-year-old woman that we are reporting on had many of the radiologic findings reported in the literature, which confirms that these features can be used to aid in the diagnosis of this rare cancer. The addition of this case report to the growing literature pool surrounding this disease can help clinicians in the future to keep this diagnosis on their differential diagnosis, ultimately providing the greatest benefit to the patient.

## Conclusions

Radiologic studies can help aid in the diagnosis of spindle cell carcinoma of the breast. A rapidly growing, solitary mass in the breast that appears circumscribed and irregular with cystic components and posterior acoustic enhancement on ultrasound, lacks calcifications on mammography, and shows central necrosis on MRI should raise suspicion for this diagnosis. This case adds to the growing body of literature surrounding this rare, aggressive breast malignancy and will hopefully equip physicians with the knowledge to make a prompt diagnosis and improve patient outcomes.
